# Improved L_0_ Gradient Minimization with L_1_ Fidelity for Image Smoothing

**DOI:** 10.1371/journal.pone.0138682

**Published:** 2015-09-18

**Authors:** Xueshun Pang, Suqi Zhang, Junhua Gu, Lingling Li, Boying Liu, Huaibin Wang

**Affiliations:** 1 School of Computer Science and Communication Engineering, TJUT, Tianjin, China; 2 School of Information Engineering, Tianjin University of Commerce, Tianjin, China; 3 The Key Lab of Big Data Computing of Hebei Province, School of Computer Science and Engineering, HEBUT, Tianjin, China; Chongqing University, CHINA

## Abstract

Edge-preserving image smoothing is one of the fundamental tasks in the field of computer graphics and computer vision. Recently, L_0_ gradient minimization (LGM) has been proposed for this purpose. In contrast to the total variation (TV) model which employs the L_1_ norm of the image gradient, the LGM model adopts the L_0_ norm and yields much better results for the piecewise constant image. However, as an improvement of the total variation (TV) model, the LGM model also suffers, even more seriously, from the staircasing effect and is not robust to noise. In order to overcome these drawbacks, in this paper, we propose an improvement of the LGM model by prefiltering the image gradient and employing the L_1_ fidelity. The proposed improved LGM (ILGM) behaves robustly to noise and overcomes the staircasing artifact effectively. Experimental results show that the ILGM is promising as compared with the existing methods.

## Introduction

Image smoothing aims at removing the insignificant details and preserving salient structure such as edges, there are many applications of image smoothing in computer graphics and image processing. However, there is always a dilemma for the smoothing algorithms to simultaneously remove details and preserve edges. For example, the linear Gaussian filter can smooth images effectively but blur the edges seriously. The objective of almost all methods focuses on how to get a tradeoff between flattening details and preserving sharp edges and there have been a plenty of works devoted to this task. Bilateral filter is one of such works and it is simple and effective in removing noise-like structures[1]. Soon after its debut in 1998, there has been a flurry of extensions such as [2–6] and its accelerated variants [7–9]. A comprehensive review of the bilateral filter was presented in [10]. Anisotropic diffusion [11] is another category of filter aiming at suppressing noise while preventing salient edges. Since its introduction, there has been a great deal of researches devoted to the theoretical and practical understanding of this model for image smoothing [12–20]. For example, Black et al. proposed the robust anisotropic diffusion model based on robust statistics [12], Weickert proposed the structure tensor model based diffusion [13][14], Sochen et al. proposed the manifold model based diffusion [15], and modified Perona-Malik model based on directional Laplacian was proposed in [16]. The fourth-order partial differential equation models were also proposed, such as [17–20]. Total variation (TV) takes the form of an unconstrained optimization model [21], where the desired image is obtained as the minimizer to a certain functional that contains both regularization and fidelity terms. The TV model employs the L_1_ norm of the image gradient as the regularization term and behaves more stably than the anisotropic diffusion [11]. Therefore, the TV model was studied and applied widely for image smoothing [22–30]. However, the TV-based approach suffers from staircasing effect. There are also other works aiming at image smoothing, such as the local extremes model by Subr et al.[31], the guided filter model by He et al.[32] and the weighted least squares method [33].

Recently, Xu et al. proposed an improvement of the TV model by replacing the L_1_ norm of the image gradient with the L_0_ norm, i.e., the L_0_ gradient minimization (LGM)[34]. The LGM model performs more effectively than the TV model for piecewise constant images, however, just as the TV model, the LGM model also suffers, even more seriously, from the staircasing effect. Since the LGM model counts the number of non-zero gradients in the result, it is not robust to noise. In order to overcome these drawbacks of the LGM model, an improved LGM (ILGM) mode is proposed by prefiltering the image gradient during the iteration in the LGM model. The L_1_ fidelity is also adopted since it is more robust than the L_2_ one when erroneous measurements exist [35][36]. Similar to LGM [34], the alternating minimization (AM) algorithm [37] is employed for the ILGM model by introducing auxiliary variables; the AM algorithm also yields global optimal result for the ILGM model. Experiment and comparison show that the ILGM model outperforms the LGM model on staircase suppression and noise robustness.

The rest of paper is organized as follows. Section 2 reviews the L_0_ gradient minimization (LGM), Section 3 introduces the improved L_0_ gradient minimization (ILGM) with L_1_ fidelity. In section 4, some experimental results are reported and analyzed. Finally, we conclude our work in Section 5.

## LGM: L_0_ Gradient Minimization

The LGM model [34] employs an L_0_ penalty term to directly measure gradient sparsity and has a strong ability to preserve edges. Let *f* be the input image and *U* the smoothed one, the gradient of image *U* at pixel *p* is denoted by ∇*U*_*p*_ = (∂_*x*_ *U*_*p*_,∂_*y*_ *U*_*p*_)^*T*^. L_0_ gradient measure is expressed as:

$$E(U)=\#\left\{p|\;\left|\partial_{x}U_{p}\right|+\left|\partial_{y}U_{p}\right|\neq 0\right\}\;,$$ (1)

Where #{} denotes the number of pixels of which the gradient is not zero. The term *E(U)* serves as the regularization term and is combined with a general data constraint term that makes the result *U* be structurally similar to the input image *f*. The energy function of the LGM is defined as follows:

$$\underset{U}{\text{min}}\{\sum_{p}{\left(U_{p}-f_{p}\right)}^{2}+\lambda \cdot E(U)\}\;,$$ (2)

where *λ* is a non-negative parameter directly controlling the weight of the regularization term. However, this regularization term is difficult to optimize because the value of *E(U)* may range from tens to thousands and it is non-convex and non-derivative. To solve the problem, the AM algorithm [37] is employed and two auxiliary variables *h*_*p*_ and *v*_*p*_ are introduced to replace the ∂_*x*_ *U*_*p*_ and ∂_*y*_ *U*_*p*_ respectively. The specific objective function is expressed as

$$\underset{U,h,v}{\text{min}}\{\sum_{p}{\left(U_{p}-f_{p}\right)}^{2}+\lambda \cdot E(h,v)+\beta \cdot ({\left(\partial_{x}U_{p}-h_{p}\right)}^{2}+{\left(\partial_{y}U_{p}-v_{p}\right)}^{2})\}\;,$$ (3)

Where ∂_*x*_ *U*_*p*_ and ∂_*y*_ *U*_*p*_ are replaced by *h*_*p*_ and *v*_*p*_ respectively in *E(U)*, and *β* is a parameter to control the similarity between the auxiliary variables and their corresponding gradients.

## ILGM: Improved L_0_ Gradient Minimization

Keeping in mind that the energy function for the LGM model is reformulated as Eq (3) by introducing two auxiliary variables *h* and *v*. *h* and *v* are then estimated from the image gradient (∂_*x*_ *U*,∂_*y*_ *U*) by exerting an L_0_ norm constraint. However, when there is false gradient in the image caused by noise or inhomogeneity, *h* and *v* will deviate from the correct values. Based on this consideration, we propose to pre-filter the image gradient (∂_*x*_ *U*,∂_*y*_ *U*) by an edge-preserving filter when estimating *h* and *v*. Since the L_1_ fidelity is more robust to outliers than the L_2_ one, we also adopt the L_1_ fidelity. As a result, the proposed ILGM model is formulated as follows,

$$\underset{U,h_{M},v_{N}}{\text{min}}\{\sum_{p}\left|U_{p}-f_{p}\right|+\lambda \cdot E(h_{M},v_{N})+\beta \cdot ({\left(\partial_{x}U_{p}-h_{M}{}_{p}\right)}^{2}+{\left(\partial_{y}U_{p}-v_{N}{}_{p}\right)}^{2})\}$$ (4)

Where the (*h*_*M*_, *v*_*N*_) is estimated from an optimized version (*M*,*N)* of the image gradient (∂_*x*_ *U*,∂_*y*_ *U*) when solving Eq (4). The LGM model is employed to calculate (*M*,*N*) from (∂_*x*_ *U*,∂_*y*_ *U*) as follows,

$$E_{M}=\int_{\Omega }{\left|\nabla M\right|}_{L_{0}}\;d\Omega +\lambda \cdot \int_{\Omega }{\left(M-\partial_{x}U\right)}^{2}\;d\Omega$$ (5)

$$E_{N}=\int_{\Omega }{\left|\nabla N\right|}_{L_{0}}\;d\Omega +\lambda \cdot \int_{\Omega }{\left(N-\partial_{y}U\right)}^{2}\;d\Omega$$ (6)

In order to handle the L_1_ fidelity, the variable *W* is introduced to denote the difference between *U* and *f*. Then, the functional in Eq (4) is rewritten as follows,

$$\underset{U,h_{M},v_{N},W}{\text{min}}\{\sum_{p}\alpha \cdot {\left(U_{p}-f_{p}-W_{p}\right)}^{2}+\left|W_{p}\right|+\lambda \cdot E(h_{M},v_{N})+\beta \cdot ({\left(\partial_{x}U_{p}-h_{M}{}_{p}\right)}^{2}+{\left(\partial_{y}U_{p}-v_{N}{}_{p}\right)}^{2})\}$$ (7)

The numerical implementation of Eqs (5) and (6) is identical to the LGM model, so the alternating minimization algorithm [37] is employed by introducing other auxiliary variables to approximate ∇*M* and ∇*N* respectively. As for Eq (7), it will be split into three subproblems to alternatively minimize *W*, (*h*_*M*_,*v*_*N*_) and *U* by using the AM algorithm[37], which lead to an global optimization procedure.

### *Subproblem 1*: Fixing *U*, *h*_*M*_, *v*_*N*_, solve *W*

With *U*, *h*_*M*_, *v*_*N*_ fixed, we rewrite problem (7) as

$$\underset{W}{\text{min}}\{\sum_{p}\alpha \cdot {\left(U_{p}-f_{p}-W_{p}\right)}^{2}+\left|W_{p}\right|\}$$ (8)

Through the matrix calculus, we can get the unique solver, and the solution can be expressed as,

$$W=\text{max}\{\left|U_{p}-f_{p}\right|-\frac{1}{2\alpha },0\}\frac{U_{p}-f_{p}}{\left|U_{p}-f_{p}\right|}$$ (9)

where the convention 0·(0/0) = 0 is followed, and (*U*_*p*_ − *f*_*p*_)/|*U*_*p*_ − *f*_*p*_| denotes the sign function. *α* is the parameter and set to 0.1 in our experiments.

### *Subproblem 2*: Fixing *U* and *W*, solve (*h*_*M*_,*v*_*N*_)

Given *U and W*, we first estimate *M* and *N* from (∂_*x*_ *U*,∂_*y*_ *U*) using Eqs (5) and (6) respectively. Then, the objective function for (*h*_*M*_,*v*_*N*_) reads

$$\underset{h_{M},v_{N}}{\text{min}}\{\sum_{p}\lambda \cdot E(h_{M},v_{N})+\beta \cdot ({\left(M_{p}-h_{Mp}\right)}^{2}+{\left(N_{p}-v_{Np}\right)}^{2})\}$$ (10)

where *E*(*h*_*M*_, *v*_*N*_) denotes the number of non-zero elements in *|h*_*M*_ *| + |v*_*N*_ *|*. This problem is similar to Subproblem 2 in the numerical implementation of LGM model[34], and the strategy there is employed to obtain (*h*_*M*_,*v*_*N*_). After some manipulation, the estimate of (*h*_*M*_, *v*_*N*_) is given by

(hMp,vNp)={(0,0)(Mp)2+(Np)2<λβ(Mp,Np)otherwise. (11)

In this step, we compute for each pixel *p* the minimum energy value.

### *Subproblem 3*: Fixing *W*, *h*_*M*_ and *v*_*N*_, solve *U*

Given *h*_*M*_, *v*_*N*_, *W*, the objective function for *U* reads

$$\underset{U}{\text{min}}\{\sum_{p}\alpha \cdot {\left(U_{p}-f_{p}-W_{p}\right)}^{2}+\beta \cdot ({\left(\partial_{x}U_{p}-h_{Mp}\right)}^{2}+{\left(\partial_{y}U_{p}-v_{Np}\right)}^{2})\}$$ (12)

Eq (12) is quadratic and thus has a global minimum even by gradient decent. Alternatively, it can be efficiently solved using Fast Fourier transform (FFT), which yields solution

$$U={\text{F}}^{-1}(\frac{\alpha \cdot (\text{F}(f)+\text{F}(W))+\beta \cdot ({\text{F}}^{*}(\partial_{\text{x}})\text{F}(h_{M})+{\text{F}}^{*}(\partial_{\text{y}})\text{F}(v_{N}))}{\alpha \cdot \text{F}(1)+\beta \cdot ({\text{F}}^{*}(\partial_{\text{x}})\text{F}(\partial_{x})+{\text{F}}^{*}(\partial_{\text{y}})\text{F}(\partial_{y}))})$$ (13)

where F(·) is the FFT operator and F(·)* denotes the complex conjugate.

The whole algorithm is summarized in Algorithm 1. Parameter *β* is automatically adapted in iterations starting from a small value *β*_0_, it is multiplied by *κ* each time.

**Algorithm 1:** ILGM: Improved L_0_ Gradient Minimization.

***Input*:** image *f*, *λ*, *β*_max_, *α*, and *κ*
***Initialization*:** *U = f*, the current parameter *β*_0_ = *λ*, *α* = 0.1, *κ* = 2, *β*_max_ = 1.0*e*5, *i* = 0
***While*** *β* < *β*_max_ **, *do***
With *U* ^(*i*)^, solve for *W* ^(*i*)^ in Eq (9)
With *U* ^(*i*)^, solve for *M* ^(*i*)^ and *N* ^(*i*)^ in Eqs (5) and (6)
With *M* ^(*i*)^ and *N* ^(*i*)^, solve for $h_{M}^{\left(i\right)}$, $v_{N}^{\left(i\right)}$ in Eq (11)
With $h_{M}^{\left(i\right)}$, $v_{N}^{\left(i\right)}$ and *W* ^(*i*)^, solve for *U* ^(*i*+1)^ in Eq (13)
Update the parameter *β* = *κ* ⋅ *β*, *i*++
***End***
***Output*:** The smoothed image *U*

## Experimental Results

In this section, we will demonstrate the performance of the proposed method and make a comparison with several state-of-the-art methods including Bilateral filtering(BLF) [1], Weighted least square method(WLS)[33] (http://www.cs.huji.ac.il/~danix/epd/wlsFilter.m), Total variation (TV)[21] (The minimization of TV is solved using Chambolle's method in Ref.[29], when a color image is encountered, a generalization of Chambolle's method in Ref.[30] is employed.), and the LGM model [34]. Several natural images are adopted as test images, and we focus on the performance of noise robustness, edge preservation, and staircasing effect. To get the best results, the parameters for these methods are manually tuned and presented in the figure captions.

**Fig 1:**
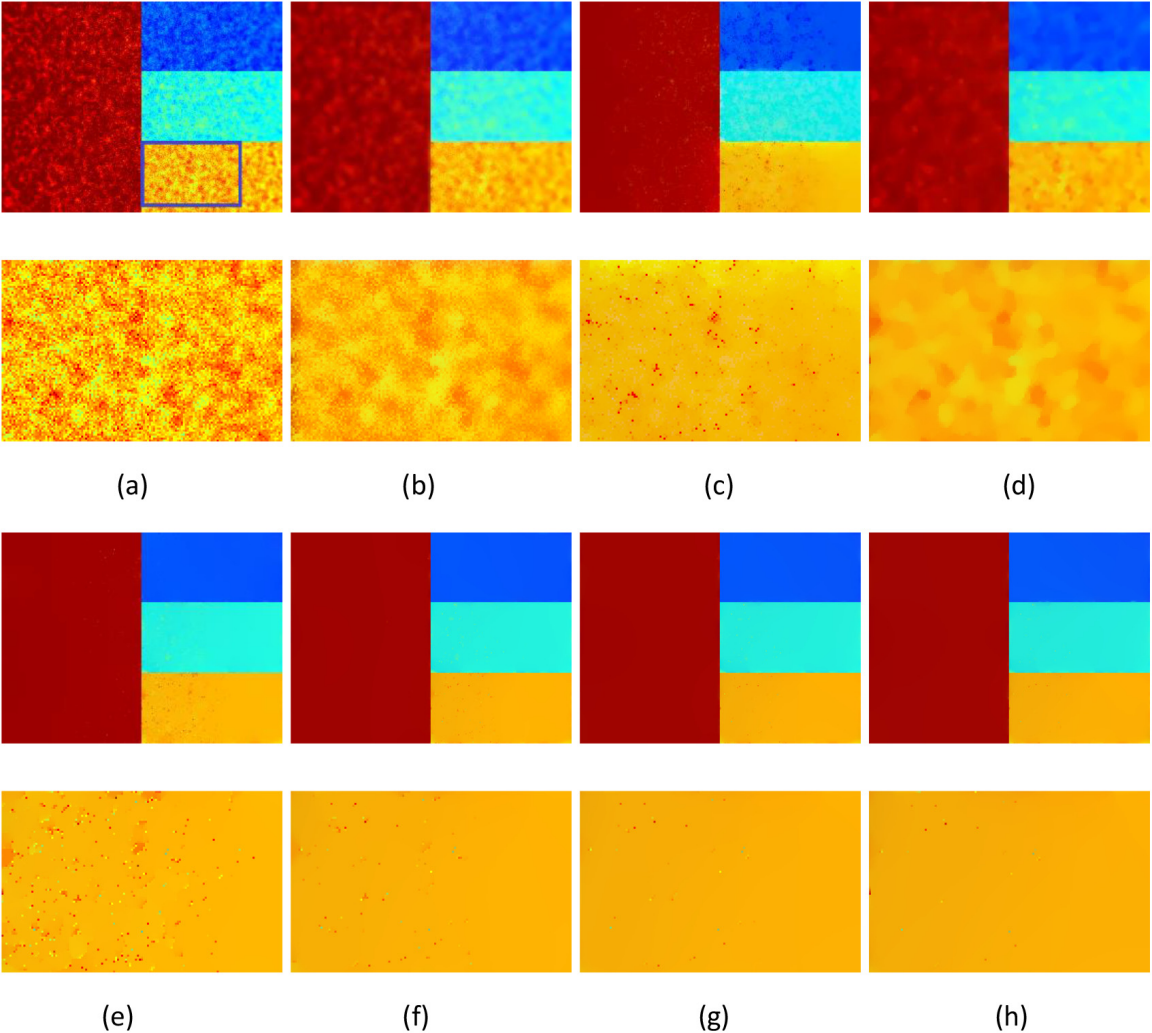
Comparison of the ILGM method with the BLF,WLS, TV and LGM methods on a synthetic image. (a) Noisy image, (b) BLF (*σ*_*s*_ = 12, *σ*_*r*_ = 0.5), (c) WLS (*λ* = 2, *α* = 3), (d) TV (*λ* = 3), (e) LGM (*λ* = 0.3), (f) ILGM (*λ* = 0.004), (g) ILGM (*λ* = 0.005) and (h) ILGM (*λ* = 0.006). Images in 2nd and 4th row are the local close-ups of those in 1st and 3rd row.

**Fig 2:**
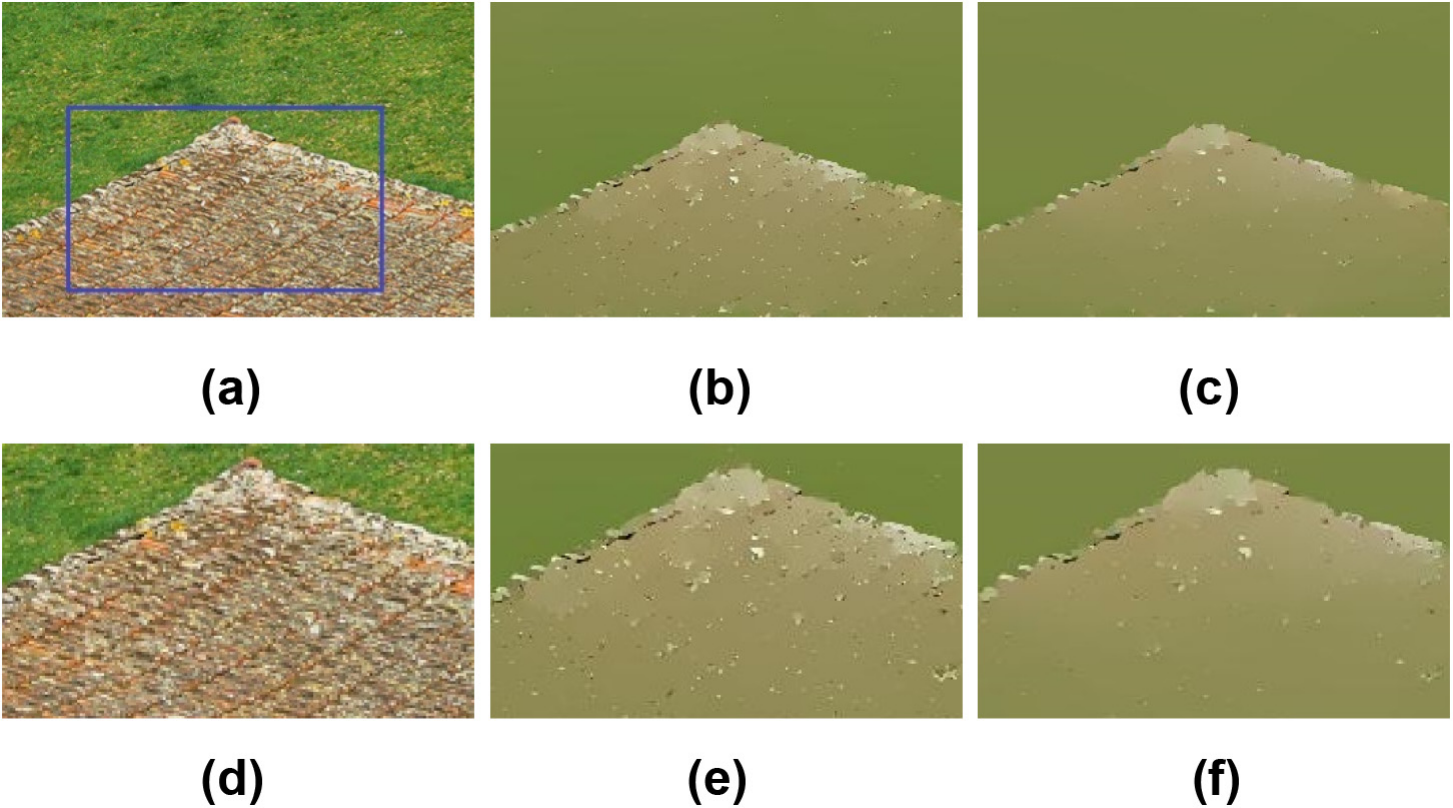
Smoothing results of a natural image using the LGM and ILGM. (a) A natural image, results of (b) LGM (*λ* = 0.3), and (c) ILGM (*λ* = 0.005). (d)-(f) are the close-ups of (a)-(c) marked by blue rectangle in (a).

The noisy color image coined by Farbman et al. [33] is employed to test the noise robustness of the proposed ILGM model. The results are presented in Fig 1. Since a prefiltering process is employed for the ILGM, the *λ* is smaller than that for the original LGM model. However, the proposed ILGM model is not sensitive to *λ*. For example, Fig 1F, 1G and 1H are the results with different *λ*, which are almost indistinguishable. Since the numerical implementation or parameters of the associated methods are not presented in [34], the results in Fig 1 are slightly different from those in [34], however, similar results are reported in [36]. Fig 2 shows another example. In this example, the ILGM method also yields a much better result. These observations demonstrate the noise robustness of ILGM method.

**Fig 3:**
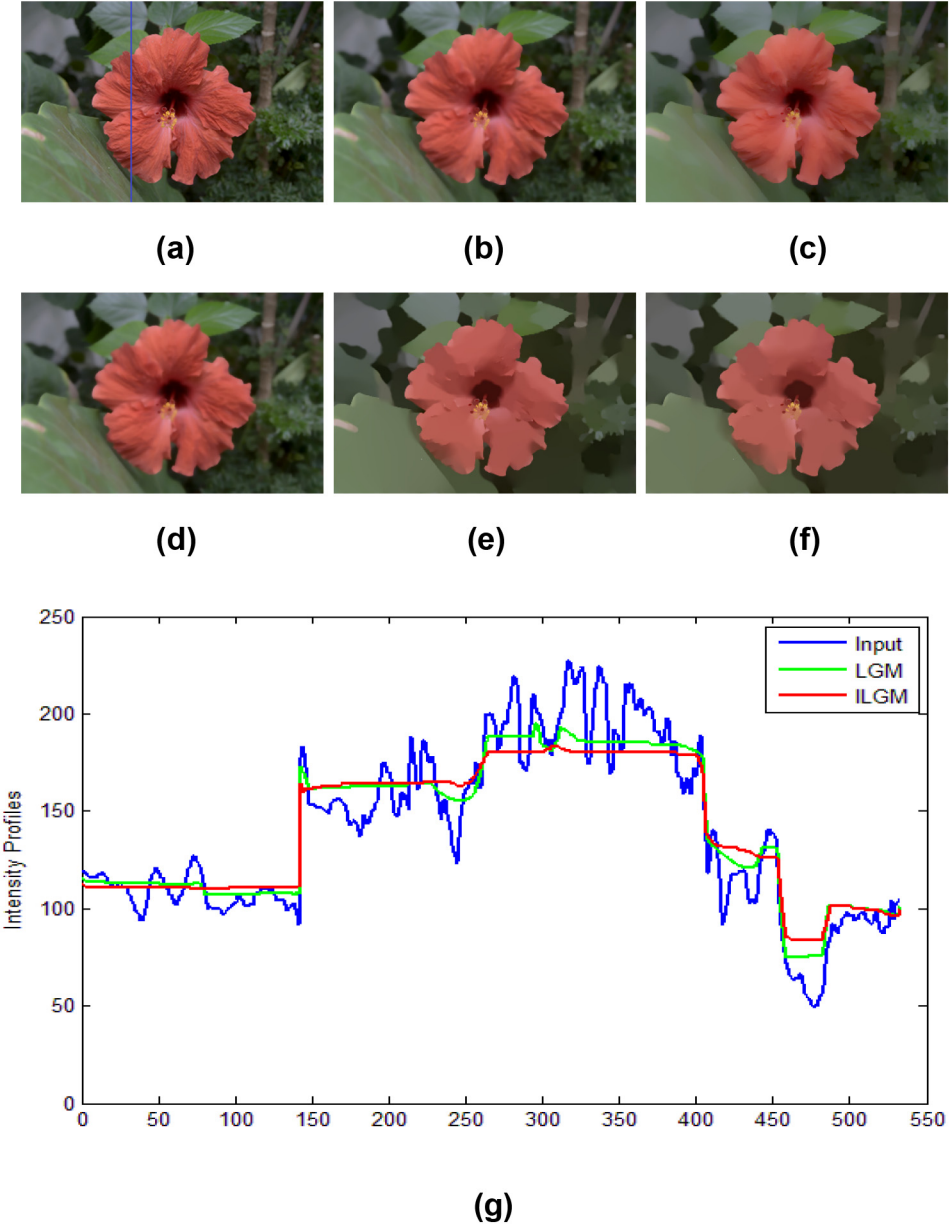
Results of image smoothing on a flower image. (a) flower image, results by (b) BLF(*σ*_*s*_ = 4, *σ*_*r*_ = 0.2), (c) WLS(*λ* = 0.4, *α* = 1.4), (d) TV(*λ* = 3), (e) LGM(*λ* = 0.02), and (f) ILGM(*λ* = 0.0006). (g) Line profiles of the R channel of the 291st column (blue line in (a)).

Fig 3 shows a flower image. In this example, we aim at smoothing the texture in the leaves and flower petals, but preserving the sharp edge between the leaf and the petal. The results of the BLF, WLS, TV, LGM, and ILGM models are presented. One can see from Fig 3 that there is still texture in the results of the BLF, WLS and TV models, and the results of the LGM and ILGM models are approximately piecewise constant. However, from the line profiles shown in Fig 3G, it is clear that there are still oscillations in the results of the LGM model. In contrast, the result of the ILGM model is much nearer to piecewise constant but the sharp edges are also preserved.

**Fig 4:**
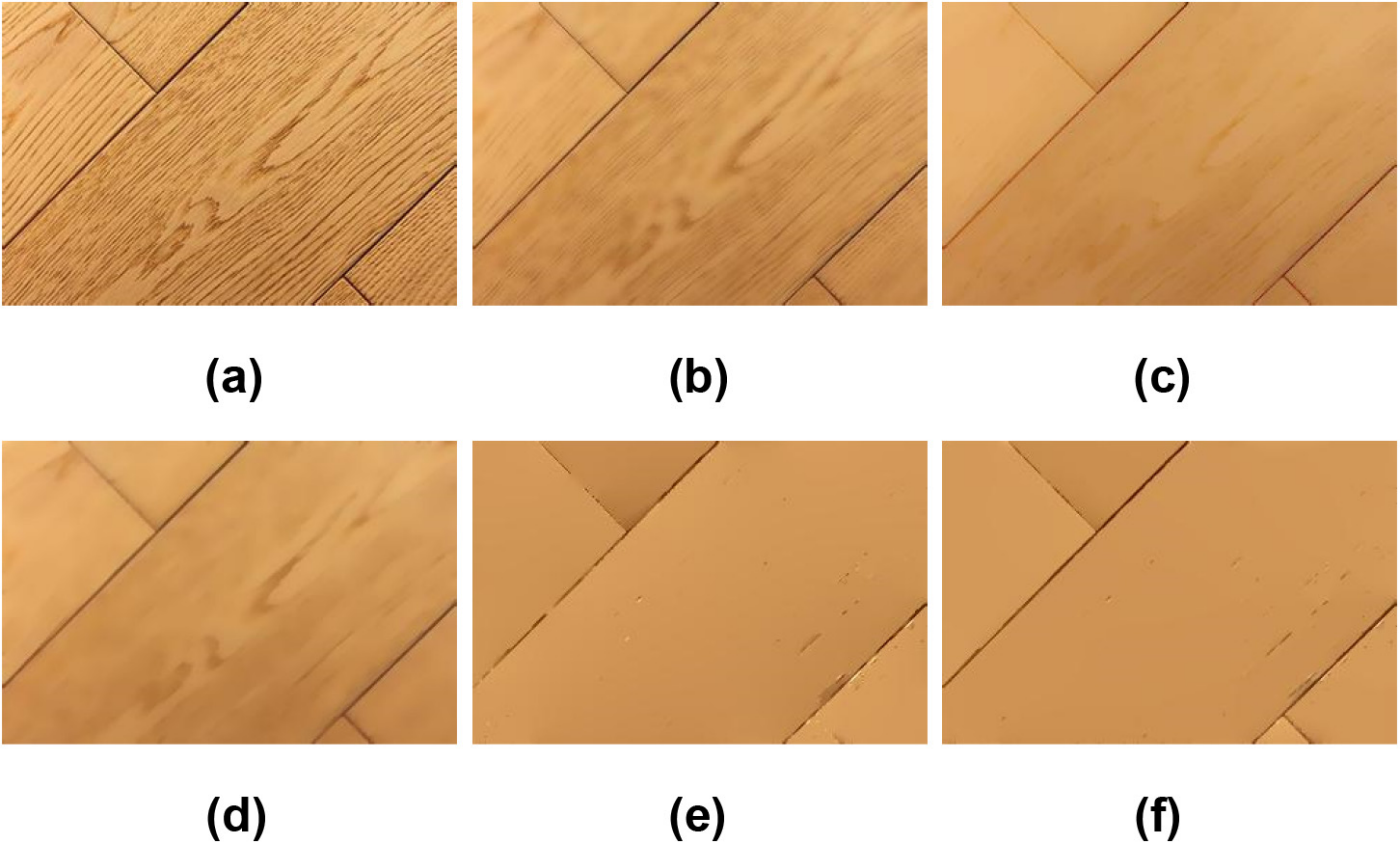
Edge-preserving experiment on a wood image. (a) The original image, results by (b) BLF (*σ*_*s*_ = 4, *σ*_*r*_ = 0.2), (c) WLS (*λ* = 0.8, *α* = 2), (d) TV(*λ* = 4), (e) LGM (*λ* = 0.3), and (f) ILGM(*λ* = 0.0025).

**Fig 5:**
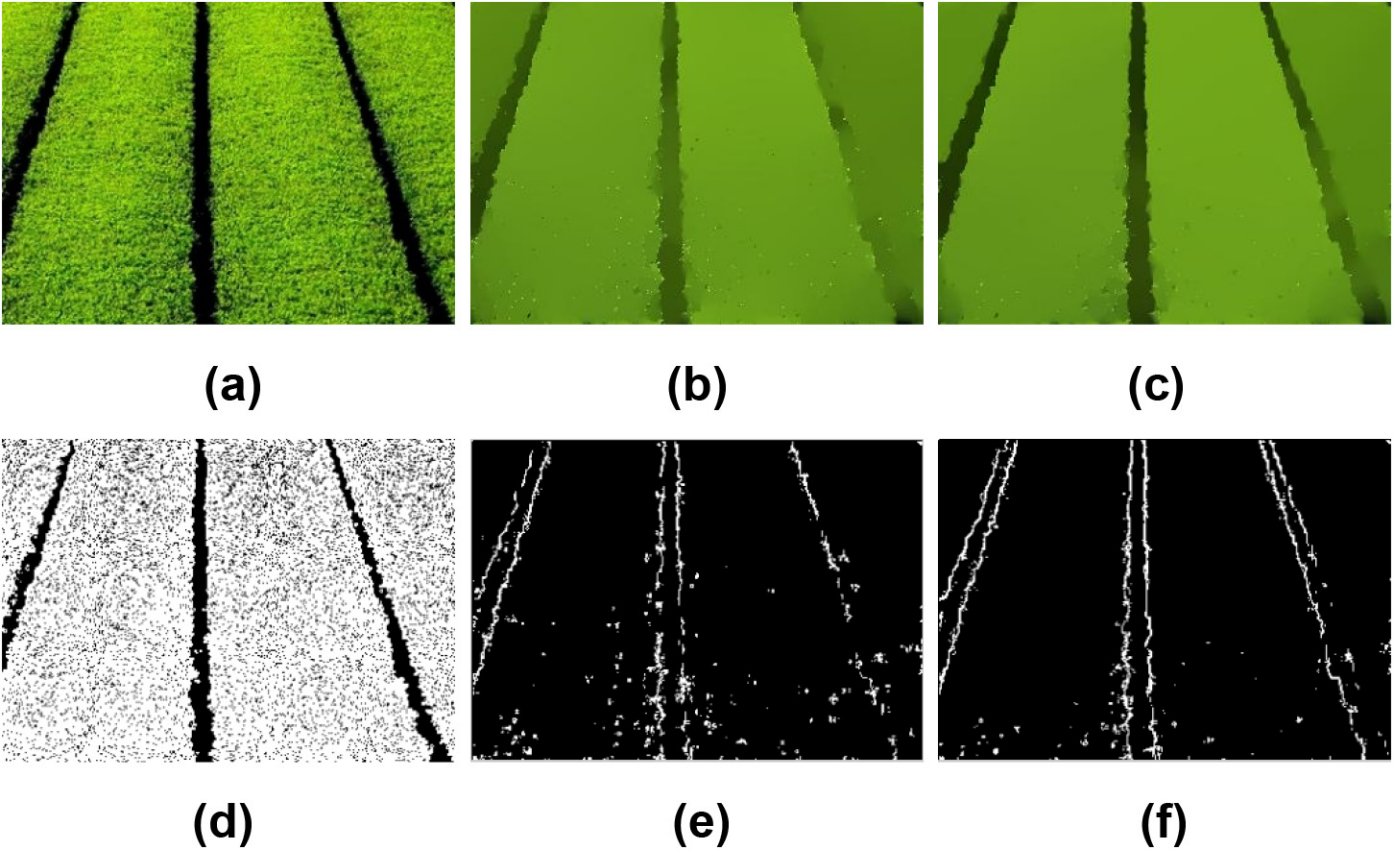
Comparison of edge-preserving performance on a natural image. (a) The natural image, results by (b) LGM (*λ* = 0.35), and (c) ILGM(*λ* = 0.0035). (d)-(f) are the edge detection results of (a)-(c), respectively.

Figs 4 and 5 show another two examples to demonstrate the edge preservation performance of the associated methods. It is clear from Fig 4 that there is still texture in the results of the BLF, WLS and TV methods, even though the edges in (c) and (d) are somewhat blurred. The LGM model smoothed out the texture clearly, but the edges are merged, see Fig 4E. On the other hand, the ILGM method preserved the sharp edges very well while smoothing out the texture clearly. Fig 5A shows an image of texture. In this image, we also aim at smoothing out the texture while preserving the edges. We first smoothed the image using LGM and ILGM respectively, then detected the edges in the results. The smoothed images and edge detection results are shown in Fig 5. One can conclude that the ILGM model performs much better than the LGM model from this example.

**Fig 6:**
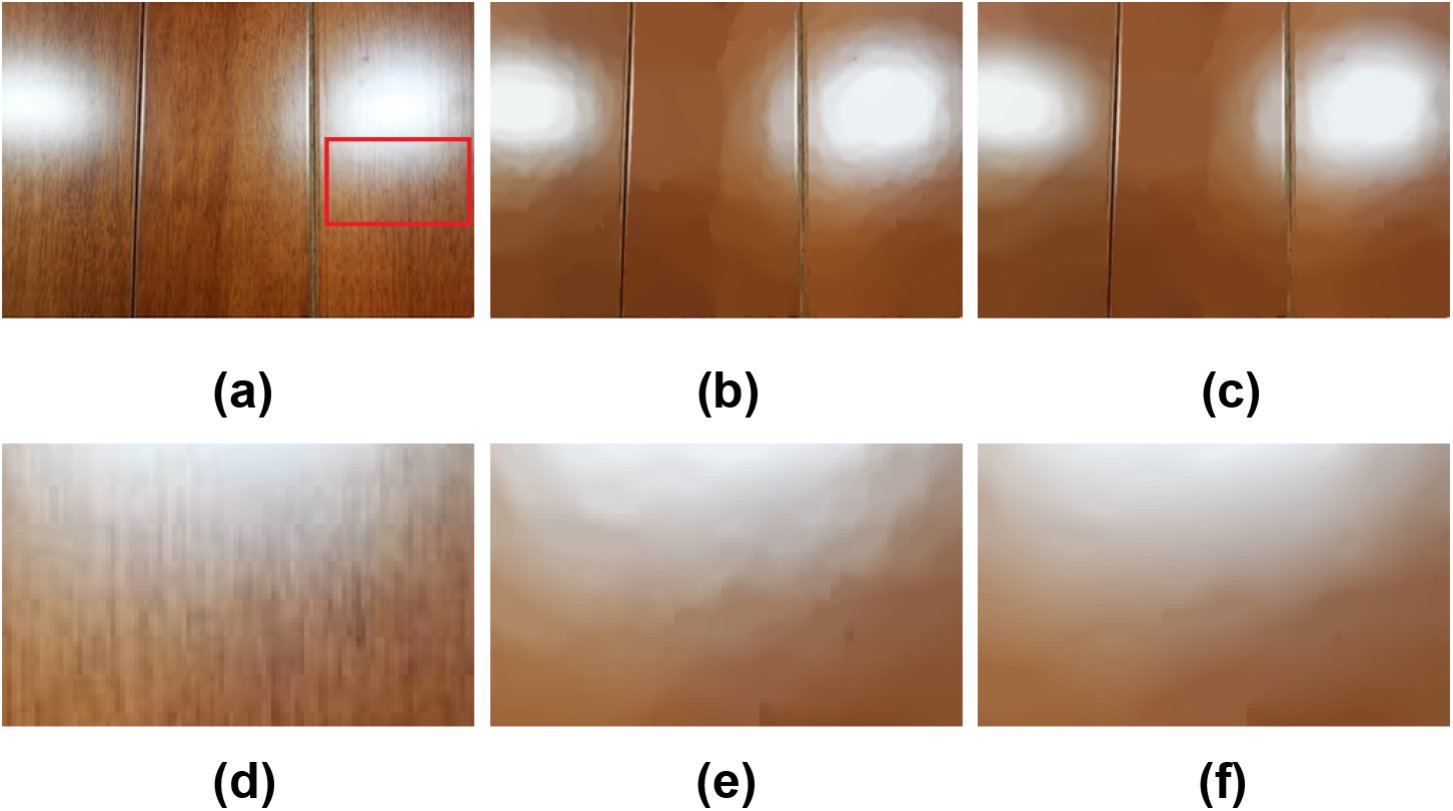
Comparison of the staircasing effect of the LGM and ILGM models. (a) The wood image, results by (b) LGM (*λ* = 0.007), and (c) ILGM (*λ* = 0.0002). (d)-(f) are the close-ups of (a)-(c),respectively.

It is well-known that the TV model suffers from the staircasing effect [21][23]. Although the LGM model replaces the L_1_ norm in the TV model with the L_0_ norm, it also suffers from this notorious effect [34]. However, the proposed ILGM model can conquer this staircasing effect effectively. Fig 6 shows an example of this case. In this example, the wood texture image suffers from inhomogeneity. When smoothing the texture, the LGM model yields staircases due to intensity inhomogeneity, see (b) and the close-up (e). However, the proposed ILGM model yields a much smoother result, see (c) and the close-up (f), and the wood texture is also removed. This example manifests that the ILGM model outperforms the LGM model on staircase suppression.

## Conclusion

Recently, the L_0_ gradient minimization (LGM) is proposed for image smoothing and performs effectively for piecewise constant images. However, it suffers from staircasing effect and is not robust to noise. In this paper, we presented an improved LGM (ILGM) model by prefiltering the image gradient and employing L_1_ fidelity. The proposed ILGM model behaves robustly to noise and overcomes the staircase artifact effectively. Experimental results and comparison with several state-of-the-art methods show that the ILGM model is promising. In the future, we will expand our method to find more applications.

## References

[1] Tomasi C, Manduchi R. Bilateral filtering for gray and color images. In ICCV.1998. p. 839–846.

[2] Choudhury P, Tumblin J. The trilateral filter for high contrast images and meshes. Eurographics Symposium on Rendering. 414:2003. p. 1–11

[3] Fattal R. Edge-avoiding wavelets and their applications. ACM Transactions on Graphics.2009; 28(3): 22.

[4] Baek J, Jacobs D E. Accelerating spatially varying Gaussian filters. ACM Transactions on Graphics. 2010; 29(6): 169.

[5] Kass M, Solomon J. Smoothed local histogram filters. ACM Transactions on Graphics. 2010; 29(4): 100.

[6] Su Z, Luo X, Deng Z, Liang Y, Ji Z. Edge-Preserving Texture Suppression Filter Based on Joint Filtering Schemes, IEEE trans Multimedia, 2013,15(3)535–548

[7] Paris S, Durand F. A fast approximation of the bilateral filter using a signal processing approach. Computer Vision-ECCV; 2006 p. 568–580.

[8] Weiss B. Fast median and bilateral filtering, ACM Transactions on Graphics.2006; 25(3): 519–526.

[9] Chen J, Paris S, Durand F. Real-time edge-aware image processing with the bilateral grid, ACM Transactions on Graphics.,2007; 26(3):103

[10] Paris S, Kornprobst P, Tumblin J, Durand F. Bilateral filtering: Theory and application, Found. Trends Comput. Graphics Vis., 2009, 4(1)1–73.

[11] Perona P, Malik J. Scale-space and edge detection using anisotropic diffusion. IEEE trans Pattern Analysis and Machine Intelligence. 1990; 12(7): 629–639.

[12] Black MJ, Sapiro G, Marimont D H, Heeger D. Robust anisotropic diffusion. IEEE trans Image Processing. 1998; 7(3): 421–432. 10.1109/83.661192 18276262

[13] Weickert J. Coherence-enhancing diffusion of color images, Image and Vision Computing, 1999, 17 (3) 201–212

[14] Weickert J. Coherence-enhancing diffusion filtering, International Journal of Computer Vision, 1999, 31(1)111–127

[15] Sochen N, Kimmel R, Malladi R, A general framework for low level vision, IEEE trans Image Processing 1998,7 (3) 310–318. 10.1109/83.661181 18276251

[16] Wang Y, Guo J, Chen WF, Zhang W, Image denoising using modified Perona-Malik model based on directional Laplacian, Signal Processing, 2013,93(9) 2548–2558.

[17] Lysaker M, Lundervold A, Tai XC. Noise removal using fourth- order partial differential equation with applications to medical magnetic resonance images in space and time, IEEE trans Image Processing 2003,12 (12) 1579–1590. 10.1109/TIP.2003.819229 18244712

[18] You Y, Kaveh M. Fourth order partial differential equations for noise removal, IEEE trans Image Processing, 2000, 9 (10) 1723–1730. 10.1109/83.869184 18262911

[19] Hajiaboli MR. An anisotropic fourth-order diffusion filter for image noise removal, International Journal on Computer Vision, 2011, 92 (2) 177–191.

[20] Wang Y, Ren W, Wang H (2013) Convolutional Virtual Electric Field based Anisotropic diffusions for image restoration, Computers and Mathematics and Applications, 66(10) 1729–1742.

[21] Rudin LI, Osher S, Fatemi E. Nonlinear total variation based noise removal algorithms. Physica D: Nonlinear Phenomena. 1992; 60(1): 259–268.

[22] Fu S, Zhang C. Adaptive non-convex total variation regularisation for image restoration, IEE Electronics Letters, 2010, 46(13) 907–908

[23] Wang Y., Chen W., Zhou S., Yu T., Zhang Y. MTV: modified total variation model for image noise removal, IEE Electronics Letters, 2011,47(10)592–594.

[24] Fu S, Zhang C. Fringe pattern denoising via image decomposition, Optics Letters, 2012, 37(3) 422–424. doi: 10.1364/OL.37.000422 22297373 10.1364/OL.37.000422

[25] Zhang H, Wang Y. Edge Adaptive Directional Total Variation, IET the Journal of Engineering, 2013, doi: 10.1049/joe.2013.0116

[26] Chambolle A, Lions PL. Image recovery via total variation minimization and related problems. Numerische Mathematik. 1997; 76(2): 167–188.

[27] Wu CL, Tai XC. Augmented Lagrangian method, dual methods and split bregman iteration for ROF, vectorial TV, and high order models, SIAM Journal on Image Sciences, 2010,3(3)300–339.

[28] Tai XC, Wu CL. Augmented Lagrangian method, dual methods and split bregman iteration for ROF model, SSVM 2009, LNCS 5567, pp. 502–513.

[29] Chambolle A. An Algorithm for Total Variation Minimization and Applications, J. Math. Imaging and Vision, 2004, 20 (1–2): 89–97.

[30] X Bresson, TF Chan. Fast Minimization of the Vectorial Total Variation Norm and Applications to Color Image Processing, UCLA CAM Report 07–25.

[31] Subr K, Soler C, Durand F. Edge-preserving multiscale image decomposition based on local extrema. ACM Transactions on Graphics. 2009; 28(5): 147.

[32] He K, Sun J, Tang X. Guided image filtering. ECCV. 2010: 1–14.

[33] Farbman Z, Fattal R, Lischinski D, Szeliski R. Edge-preserving decompositions for multi-scale tone and detail manipulation. ACM Trans. Graph., 2008, 27, 3.

[34] Xu L, Lu C, Xu Y, Jia J. Image smoothing via L0 gradient minimization. ACM Transactions on Graphics. 2011; 30(6):174.

[35] Shen CT, Chang FJ, Hung YP, Pei SC. Edge-preserving image decomposition using L1 fidelity with L0 gradient. SIGGRAPH Asia 2012 Technical Briefs.2012: 6.

[36] Cheng X, Zeng M, Liu X. Feature-preserving filtering with L0 gradient minimization, Computers & Graphics, 2014; 38(2)150–157.

[37] Wang Y, Yang J, Yin W, Zhang Y. A new alternating minimization algorithm for total variation image reconstruction. SIAM Journal on Imaging Sciences, 2008; 1(3): 248–272.

